# Microglial exosomes alleviate intermittent hypoxia-induced cognitive deficits by suppressing NLRP3 inflammasome

**DOI:** 10.1186/s13062-023-00387-5

**Published:** 2023-06-13

**Authors:** Yaodan Zhang, Yuyang Miao, Xiangyang Xiong, Jin Tan, Zhaoli Han, Fanglian Chen, Ping Lei, Qiang Zhang

**Affiliations:** 1 Department of Geriatrics, Tianjin Geriatrics Institute, Tianjin Medical University General Hospital, Anshan Road No. 154, Tianjin, 300052 China; 2 Haihe Laboratory of Cell Ecosystem, Department of Geriatrics, Tianjin Medical University General Hospital, Tianjin, 300052 China; 3 Tianjin Medical University, Tianjin, 300052 China; 4 Tianjin Neurological Institute, Tianjin Medical University General Hospital, Tianjin, 300052 China

**Keywords:** Intermittent hypoxia, Exosomes, Cognition, NLRP3 inflammation, miR-146a-5p

## Abstract

Intermittent hypoxia is the best predictor of developing cognitive decline and Alzheimer’s disease progression in patients with obstructive sleep apnea. The nucleotide-binding oligomerization domain-like receptor 3 (NLRP3) inflammasome has been poorly studied as a regulator of neuroinflammation in cognitive impairment caused by intermittent hypoxia. As critical inflammatory cells, exosomes secreted by microglia have been found to affect the spread of pathologic proteins and neuropathology in neurodegenerative diseases. However, the effects of microglial exosomes on neuroinflammation and cognitive outcomes after intermittent hypoxia remain unclear. In this study, the role of miRNAs in microglial exosomes in improving cognitive deficits in mice exposed to intermittent hypoxia was investigated. We demonstrated that miR-146a-5p fluctuated over time in microglial exosomes of mice exposed to intermittent hypoxia for different periods of time, which could regulate neuronal NLRP3 inflammasome and neuroinflammation. In primary neurons, we found that miR-146a-5p regulated mitochondrial reactive oxygen species by targeting HIF1α, thus affecting the NLRP3 inflammasome and secretion of inflammatory factors. Similarly, further studies showed that inhibition of NLRP3 by administering overexpressed miR-146a-5p in microglial exosomes and MCC950 has improved neuroinflammation and cognitive dysfunction in mice after intermittent hypoxia. In conclusion, NLRP3 inflammasome may be a regulatory target for ameliorating cognitive impairment caused by intermittent hypoxia, and microglial exosomal miR-146a-5p may be a promising therapeutic strategy.

**Supplementary Information:**

The online version contains supplementary material available at 10.1186/s13062-023-00387-5.

## Background

Obstructive sleep apnea (OSA) is a global health problem with a high incidence and is characterized by chronic intermittent hypoxia (IH), sleep fragmentation and daytime sleepiness, which is associated with multiple comorbidities, including cardiovascular, metabolic diseases, and cognitive dysfunction [1–3], and inflammation plays an important role in the development of these comorbidities. Chronic intermittent hypoxia (CIH) can cause pathological diffusion of tau and aggravate memory impairment in Alzheimer’s disease (AD) mice [4]. Neuroinflammation was known to play a prominent role in the pathogenesis of AD [5]. And the assembly and activation of the NLRP3 inflammasome in dopamine neurons could be an important intervention direction for Parkinson’s disease treatment [6]. It has been suggested that NLRP3 inflammasome is involved in the production of systemic inflammatory response caused by intermittent hypoxia in OSA patients [7]. However, the role of NLRP3 inflammasome in cognitive dysfunction induced by intermittent hypoxia is rarely investigated.

As the major neuroimmune cells whose functions include sensing, housekeeping, and defense, microglia can maintain neuronal health and play neuroprotective roles [8]. Communication between nerve cells includes cytokines, antigen presentation, extracellular vesicles and so on. The communication between microglia and neurons is crucial in regulating complex functions that are key in regulating brain activity. Although the role of microglia in promoting inflammation impairment cannot be ignored, whether the neuronal inflammation is affected by microglia is also an interesting topic. Growing evidence has shown that extracellular vesicles (EVs) released by microglia can serve as critical mediators of signal communication between cells [9, 10]. EVs are mainly divided into two types, exosomes with sizes between 50 and 150 nm and microvesicles with diameters ranging from 50 to 500 nm (up to 1 μm) [11]. Microglia can promote the seeding of tau protein due to endocytosis and exocytosis, and exosomes may be an indispensable pathway [12]. Furthermore, studies have indicated that microglial exosomes promote the pathological progression of α-synuclein in Parkinson’s disease [13]. A large number of studies have shown that surveillant microglia [14] can improve the prognosis of neurological injury [15, 16]. So different states of microglia have different effects on neurons [17].

So far, to our knowledge, there have been no studies on the relationship between microglial exosomes and cognitive impairment after intermittent hypoxia. Therefore, it is essential to explore the effect and mechanism of microglia EVs on neurons after IH. It has been demonstrated that, according to our previous studies, microglia exosomes can affect neurodegeneration after repetitive mild traumatic brain injury [18]. The NLRP3 inflammasome inhibitor (MCC950), which crosses the blood-brain barrier, salvages brain tissue damage by regulating neuroinflammation in traumatic encephalopathy and Intracerebral Hemorrhage [19, 20]. On this basis, it is explored whether miRNA in microglia exosomes could affect NLRP3 inflammasome and neuroinflammatory response and its possible mechanism in this research, as well as the potential of inhibiting NLRP3 inflammasome in alleviating cognitive dysfunction after IH. To some extent, it can also provide a new therapeutic approach for improving IH-induced cognitive impairment.

## Results

### IH promotes NLRP3 inflammasome and neuroinflammation

**Fig. 1:**
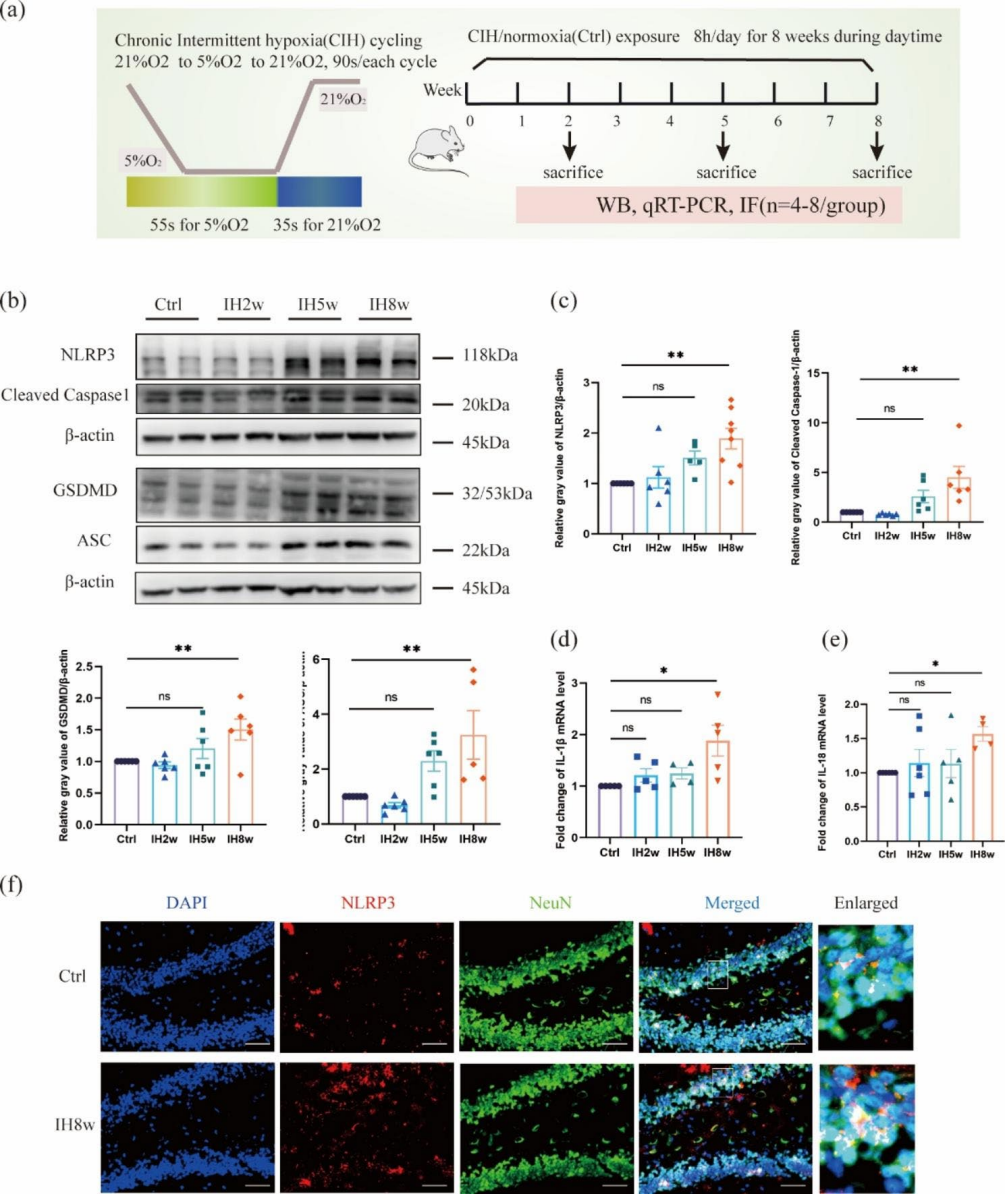
NLRP3 inflammasome and neuroinflammation were detected in mice exposed to IH8w. (**a**) Schematic diagram of experimental design, and the mouse IH cycling protocol used in this study. (**b**) Western blot was used to detect the levels of NLRP3 inflammasome proteins (NLRP3, Cleaved caspase1, GSDMD, ASC) at different time points after IH. (**c**) Protein levels were normalized to β-actin, and the results showed as fold change of control. (**d-e**) The time profiling of IL-1β, IL-18 mRNA levels in the brain of mice exposed to IH were performed by qRT-PCR. (**f**) Representative immunofluorescence staining images of NLRP3 co-localized with neurons in the dentate gyrus (DG) region of the hippocampus of mice exposed to CIH or normoxia for 8 weeks. Scale bar: 50 μm. Data are presented as mean ± SD (n = 4–8) and analyzed using one-way ANOVA with Tukey’s post hoc test. *P < 0.05, **P < 0.01, and ***P < 0.001

Previous studies have shown that NLRP3 inflammasome could drive tau pathology and aggravate cognitive impairment [21, 22]. To understand the NLRP3 inflammasome and neuroinflammatory levels at different time points of mice exposed to IH, an experimental design was conducted, as shown in Fig. 1a. We first assessed the protein levels of NLRP3, Cleaved caspase1, GSDMD, and ASC in the mice exposed to 2, 5, 8 weeks of IH or normoxia, and we found that the NLRP3 inflammasomes were activated in the brain tissue of mice exposed to CIH for 8 weeks (Fig. 1b, c). Similarly, the mRNA expression levels of inflammatory factors, including IL (interleukin) 1β and IL18 were also significantly increased in brains of mice compared to the control group (Fig. 1d-e). Next, to determine whether the activation of NLRP3 inflammasome occurs in neurons, the co-localization of NLRP3 and neurons was performed in the dentate gyrus (DG) region of the hippocampus. We found that NLRP3 expression was significantly increased in the hippocampal neurons of mice exposed to IH for 8 weeks (Fig. 1f). The NLRP3 levels were significantly elevated in mice with IH at 8 weeks compared to the control group, suggesting that neuronal inflammasome activation may play a crucial role in the nerve damage induced by IH. All these findings indicated that NLRP3 inflammasomes were activated in neurons of the mice after treating with IH for 8 weeks.

### IH induces the reactive microglia and drives tau pathology

**Fig. 2:**
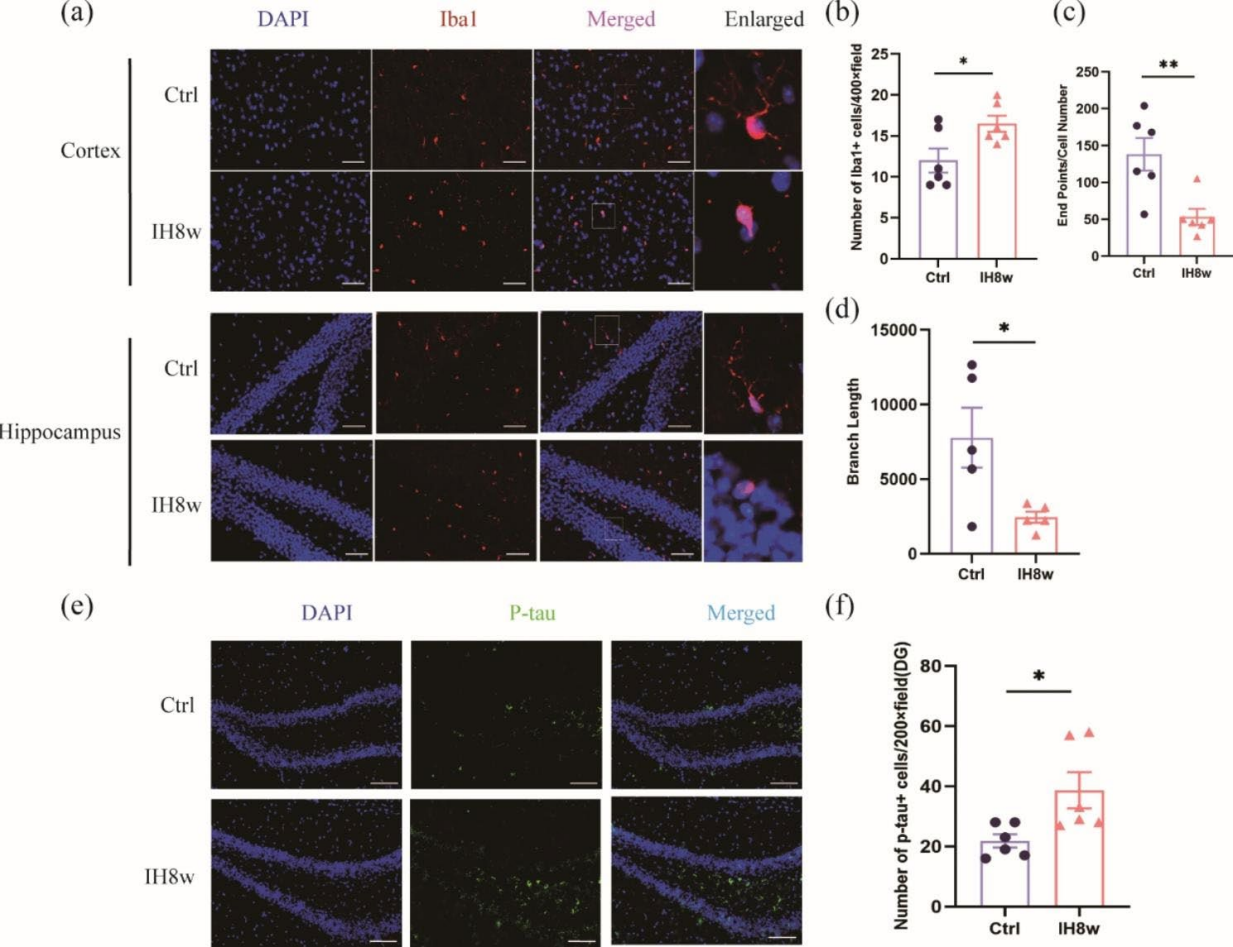
IH8w promoted microglial activation and increased p-tau expression in mice. (**a**) Representative immunofluorescence staining of Iba1 positive cells in the cortex and hippocampus of mice exposed 8 weeks to IH or normoxia. Scale bar: 50 μm. (**b**) Quantitative analysis of Iba1^+^ cells. (**c**) Endpoints of microglia were counted by Image J. (**d**) Total branch length were measured by Image J. (**e**) Representative images of p-tau and DAPI staining in the DG area of hippocampus of mice with IH for 8 weeks or normoxia. Scale bar: 100 μm. (**f**) The p-tau expression was significantly increased in the hippocampus of mice after IH8w. Data are expressed as mean ± SD and analyzed using student’s t test. *P < 0.05, **P < 0.01

It is well known that microglia may be related to the diffusion of tau pathology [23]. We further investigated that IH (8 weeks) could promote the reaction of microglia compared with the control group (Fig. 2a-d), including changes in number and morphology. Quantification of Iba1-positive cells showed a significant increase in the IH8w group (Fig. 2b). The endpoints of microglia branches were counted using Image J, and the endpoints/cell number in IH8w group was lower compared to the control group (Fig. 2c). Additionally, total branch lengths were measured using Image J, and a significant reduction was observed in the IH8w group (Fig. 2d). These findings collectively indicated that reactive microglia were induced by IH. The significant increase of p-tau protein was also observed in the hippocampus of mice (Fig. 2e-f), as shown in serum [24].

### The miR-146a-5p level in microglia and their exosomes exposed to IH

**Fig. 3:**
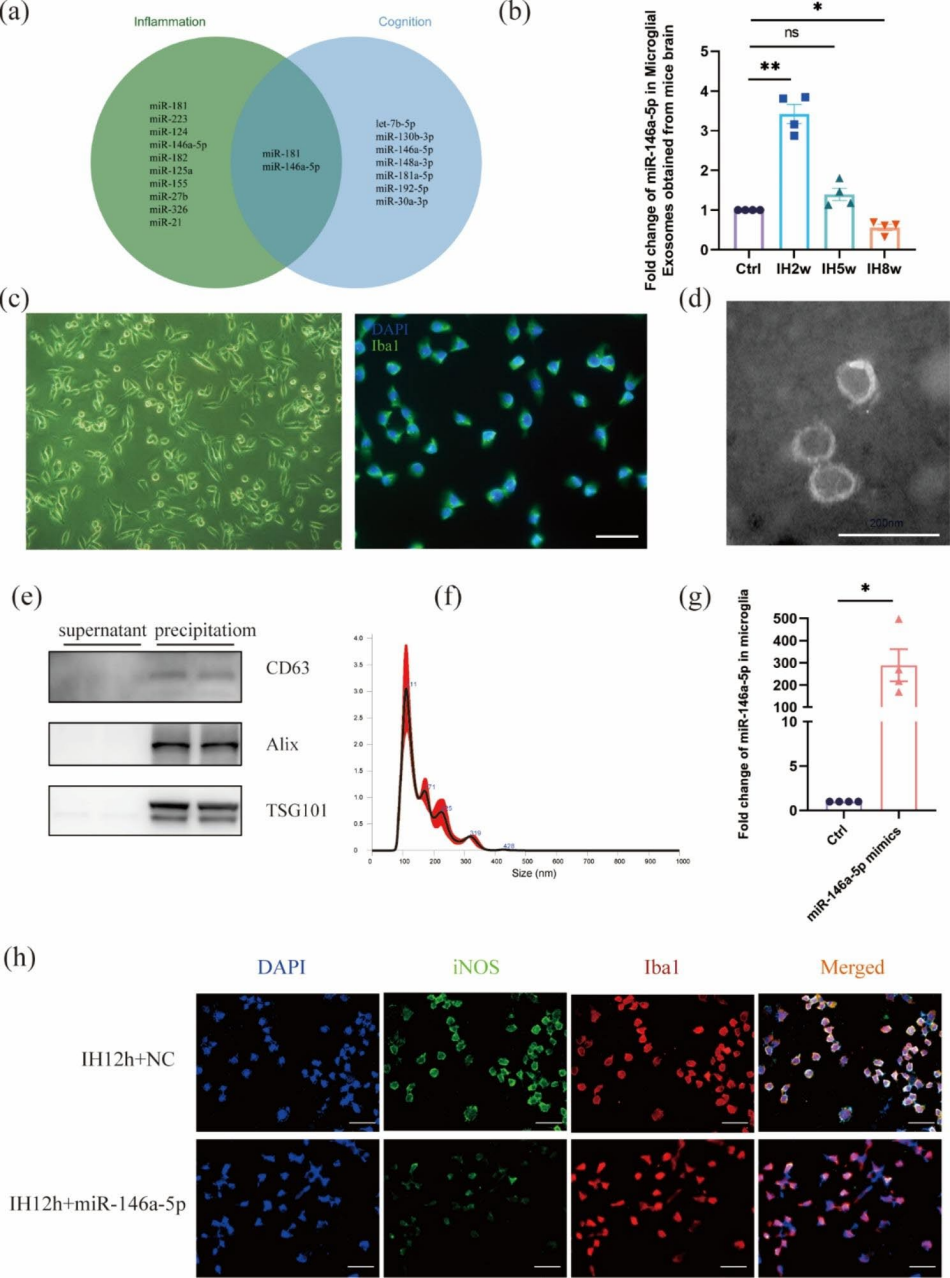
Analysis of miR-146a-5p levels in microglia and microglial exosomes after IH12h. (**a**) Venn diagram depicting miRNAs related to inflammation and cognition. (**b**) The time changes of miR-146a-5p levels in microglial exosomes acquired from mice after IH determined by qRT-PCR. (**c**) Identification of BV2 microglia using transmission light microscope and immunofluorescence staining of Iba1. Scale bar: 50 μm. (**d-f**) Identification of microglial exosomes by nanoparticle tracking analyzer, immunoblot analysis (CD63, Alix and TSG101) and transmission electron microscopy scanning. Scale bar: 200 nm. (**g**) The level of miR-146a-5p in cultured microglia after miR-124-3p mimics transfection. (**h**) IF staining showed that the number of iNOS+ /DAPI + cells in miR-124-3p mimics + IH12h group was lower than that in the IH12h + Negative control (NC) group. Scale bar: 50 μm. Data are expressed as mean ± SD and analyzed using student’s t test or one-way ANOVA with Dunnett’s T3 post hoc test. *P < 0.05, **P < 0.01

To further understand which miRNA in microglia and their exosomes can affect the level of cognition and neuroinflammation, we found the miRNA associated with inflammation [25–27] and cognition [28] through literature review (Fig. 3a). In addition, miR-146a-5p has been found to regulate NLRP3 inflammasome [29]. Next, we would like to know the miR-146a-5p levels in microglial exosomes of mice. Based on these findings, we further verified the expression change of miR-146a-5p in microglial exosomes of mice after IH. The level of miR-146a-5p increased at 2 weeks, gradually decreased to the baseline at 5 weeks, and ultimately dropped below the baseline at 8 weeks (Fig. 3b).

Next, we cultured pure BV2 microglia (Fig. 3c) and extracted exosomes from the culture medium for identification (Fig. 3d-f). TEM showed the diameter (50–150 nm) and morphology of the particles, the peak diameter of the small EVs was 111.4 ± 3.9 nm with a nanoparticle tracking analysis (NTA). In addition, the biomarkers (CD63, Alix, TSG101) were also highly expressed in the exosomes. These results indicated that exosomes were the main components of the extracted particles. As microglia with different states play different roles [14], then we wanted to know whether miR-146a-5p affected the state of microglia. The miR-146a-5p mimics was transfected into microglia, and the transfection efficiency was measured to prove the successful delivery (Fig. 3g). We found that miR-146a-5p can affect the iNOS level of microglia, indicating that the level of reactive microglia decreased after the administration of miR-146a-5p mimics (Fig. 3h). We speculated that the miR-146a-5p in microglial exosomes might be a significant factor affecting neuroinflammation after IH.

### The production of inflammatory cytokines after IH in neurons is induced by NLRP3 inflammasome

**Fig. 4:**
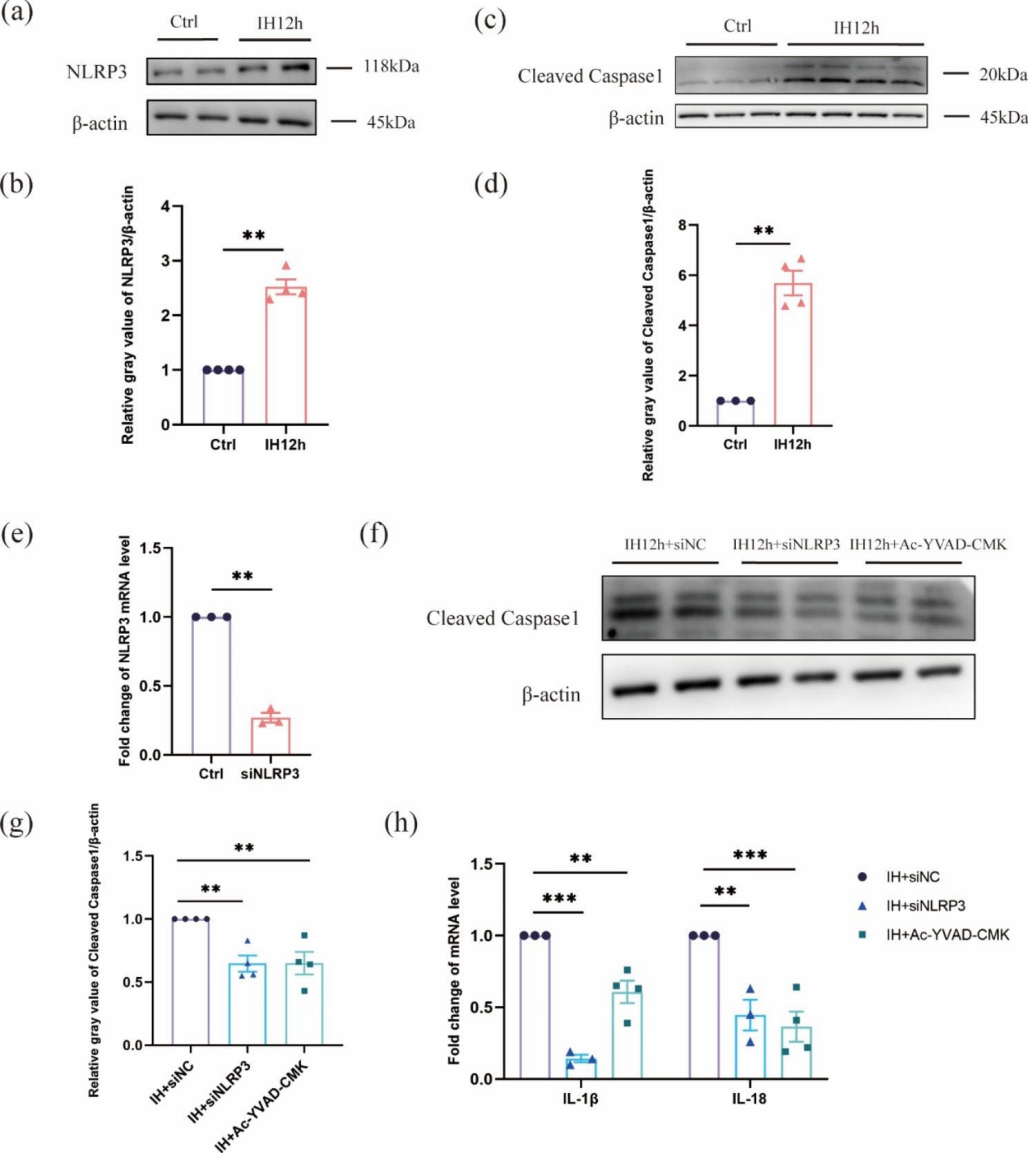
The production of inflammatory cytokine in neuron is mediated by NLRP3 inflammasome. (**a, c**) NLRP3 and cleaved caspase1 protein were measured by immunoblot after 12 h of IH in HT22 hippocampal neurons. (**b, d**) Quantitative data of NLRP3 and cleaved caspase1 protein levels after 12 h of IH in hippocampal neurons. (**e**) The expression analysis of NLRP3 mRNA by qRT-PCR was significantly decreased following siNLRP3 transfection. (**f**) The hippocampal neurons cultured with the caspase-1 inhibitor Ac-YVAD-cmk (YVAD) or transfected with siNLRP3 and then treated with IH for 12 h. The cleaved caspase1 protein were performed by western blotting. (**g**) Quantitative analysis of cleaved caspase1 in neurons. (**h**) The mRNA expression levels of IL-1β, IL-18 was detected under the above different treatment. Data are presented as mean ± SD and analyzed using student’s t test or one-way ANOVA with Tukey’s post hoc test. *P < 0.05, **P < 0.01, and ***P < 0.001. siNLRP3, a specific silencing RNA against NLRP3.

The biologically active IL -1β and IL-18 is associated with the activation of caspase-1, which leads to the cleavage of pro-IL-1β and pro-IL-18 and the eventual release of inflammatory mediators. Previous studies have shown that NLRP3 inflammasome is associated with neuroinflammation after IH by regulating mitophagy [30]. So, the activation of NLRP3 inflammasome might represent the elevation of inflammatory cytokines in the IH model. We then evaluated the NLRP3 inflammasome and cleaved caspase1 protein levels of neurons using HT22 hippocampal neurons, and found that NLRP3 and cleaved caspase1 levels elevated significantly treated with IH for 12 h (Fig. 4a-d). Subsequently, we further evaluated the role of NLRP3 in the upregulation of inflammatory cytokines. We found that mRNA levels of NLRP3 and protein levels of cleaved caspase1 were significantly decreased with siNLRP3 transfection. After pharmacological inhibition of caspase-1 (Ac-YVAD-cmk), cleaved caspase-1 was reduced (Fig. 4e-g). Our data showed a decrease of IL1β and IL-18 mRNA levels in neurons after treatment with siNLRP3 and caspase1 inhibitor (Fig. 4h). Together, these data suggest that IH promotes the secretion of inflammatory cytokines partially mediated by the NLRP3 inflammasome.

### Microglial exosomal miR-146a-5p alleviates NLRP3 inflammasome in IH neurons

**Fig. 5:**
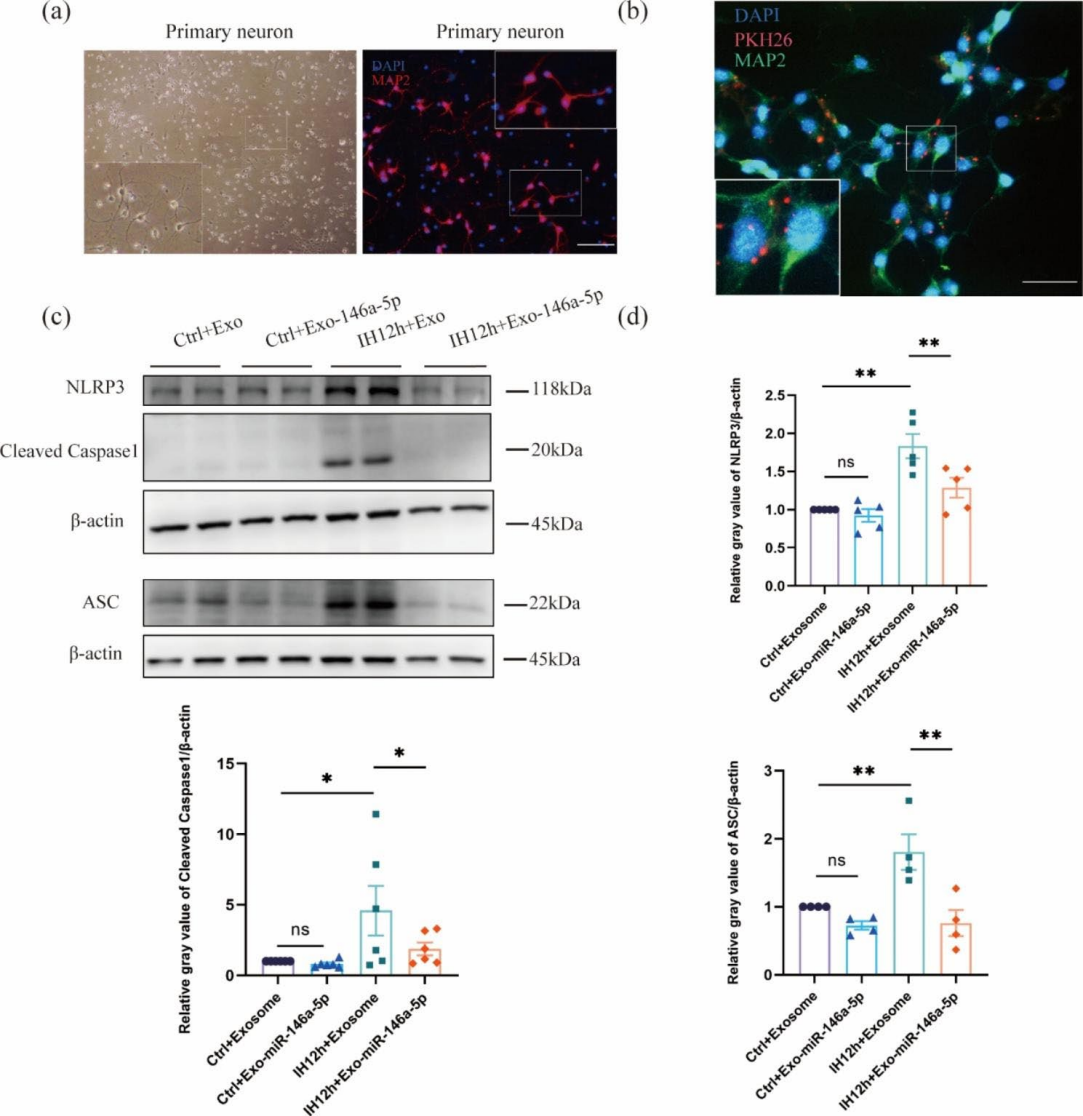
Overexpressed miR-146a-5p in microglial exosomes alleviates neuronal NLRP3 inflammasome and neuroinflammation after IH. (**a**) Primary neurons were identified by transmission light microscope and IF staining of MAP2. Scale bar: 50 μm. (**b**) Immunofluorescence staining showed that PKH26 was co- expression with MAP2, demonstrating that exogenous exosomes were taken up by neurons. Scale bar: 50 μm. (**c, d**) Western blot (**c**) and quantitative data (**d**) of NLRP3 inflammasome proteins (NLRP3, Cleaved caspase1, ASC) in primary neurons after IH and treatment with exosomes. Data are presented as mean ± SD and analyzed using one-way ANOVA with Tukey’s post hoc test. *P < 0.05, **P < 0.01, and ***P < 0.001

In order to study the effect of miR-146a-5p in microglial exosomes on the NLRP3 inflammasome after IH, we extracted the primary neurons and identified them with transmission light microscope and immunofluorescence staining of MAP2 (Fig. 5a). Then, exosomes were harvested from microglia with high miR-146a-5p expression or from unedited microglia, and added to neurons. Immunofluorescence staining showed that MAP2 and PKH26 were co-expressed in neurons, indicating that exosomes were taken up by neurons (Fig. 5b). The levels of NLRP3 inflammasome-related proteins, including NLRP3, Cleaved Caspase1, and ASC, were examined in neurons after treatment with IH and exosomes. We found that the NLRP3 inflammasome was significantly activated after IH, and the expression of NLRP3 inflammasome (NLRP3, Cleaved Caspase1, and ASC) was significantly inhibited in IH neurons treating with miR-146a-5p up-regulated exosomes compared with unedited exosomes (Fig. 5c-d). In addition, to rule out the effect of unedited exosomes on the NLRP3 inflammasome in IH neurons, we demonstrated that the activation of NLRP3 inflammasome could not be reversed by unedited exosomes by western blotting (Fig. S1a-b). In a word, miR-146a-5p inhibits the activation of NLRP3 inflammasome in IH neurons.

### miR-146a-5p inhibited neuronal inflammation by targeting the HIF1α/mtROS signaling pathway in neurons after IH

**Fig. 6:**
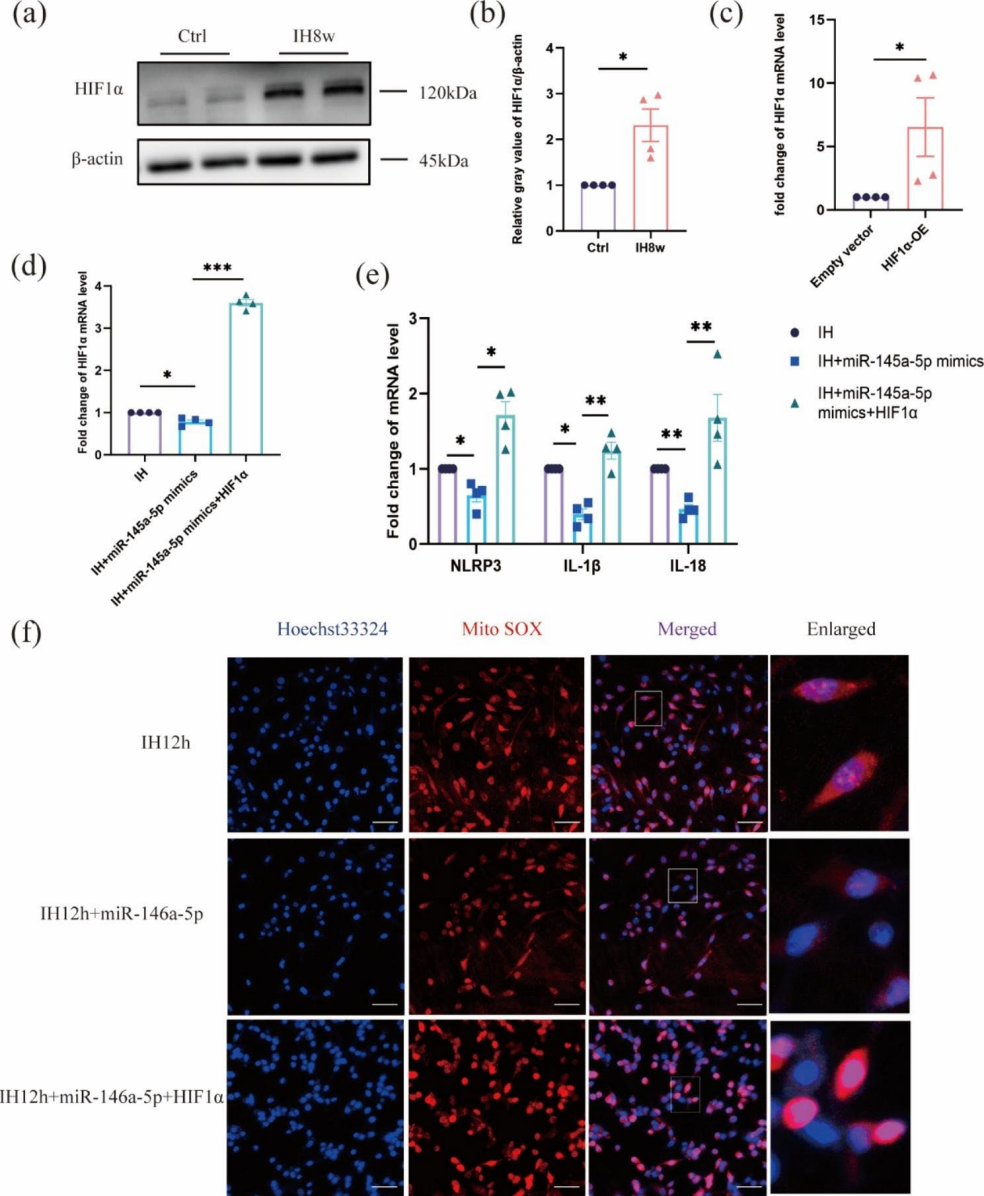
miR-146a-5p inhibits neuronal NLRP3 inflammasome in neurons after IH by targeting HIF1α and regulating mtROS expression. (**a**) Western blot for HIF1α was performed on mice exposed to IH for 8weeks. (**b**) Protein expression was normalized to β-actin and expressed as a fold change in control. Data are expressed as mean ± SD (n = 4) and analyzed using student’s t test. *P < 0.05, **P < 0.01, and ***P < 0.001. (**c**) The expression analysis of HIF1α mRNA was significantly increased after transfection of plasmids with high expression of HIF1α. Data are expressed as mean ± SD and analyzed using student’s t test. (**d-e**) The mRNA expression levels of HIF1α, NLRP3, IL-1β, IL-18 was detected in cultured neurons or culture medium of the three groups (neurons treated with IH for 12 h (IH group), neurons treated with IH for 12 h and transfected of miR-146a-5p mimics (IH + miR-146a-5p mimics group), and neurons treated with IH for 12 h, and transfected of miR-146a-5p mimics and plasmids with high expression of HIF1α (IH + miR-146a-5p mimics + HIF1α group)). Data are presented as mean ± SD and analyzed using one-way ANOVA with Tukey’s post hoc test. (**f**) The level of mitoSOX in the neurons in the three groups was observed by IF staining, which reflected the level of mtROS. Scale bar: 50 μm. Data are presented as mean ± SD and analyzed using one-way ANOVA with Tukey’s post hoc test. *P < 0.05, **P < 0.01, and ***P < 0.001

To further investigate the potential mechanism of miR-146a-5p in regulating the NLRP3 inflammasome, we conducted a thorough search of database and literature. As an important factor for disease progression in OSA patients [7, 31], HIF1α also serves as a target for miR-146a-5p [32]. Therefore, first, we demonstrated that mice exposed to IH for 8weeks increased the expression of HIF1α protein (Fig. 6a-b). Then, plasmids with high expression of HIF1α were transfected into neurons, and the transfection efficiency was obvious (Fig. 6c, Fig. S2). Subsequently, we divided the experiment into three groups and found that miR-146a-5p reduced mRNA levels of HIF1α, NLRP3, IL-1β and IL-18 of neuron after IH, which could be reversed by high expression of HIF1α (Fig. 6f). Due to the production of mtROS is related to the production of NLRP3 inflammasome activation [33]. We then explored whether miR-146a-5p could regulate mtROS and found that miR-146a-5p could down-regulate mtROS in neurons with IH, which was significantly up-regulated after HIF1α overexpression (Fig. 6g). These results showed that miR-146a-5p directly or indirectly alleviated the production of NLRP3 inflammasome and inflammatory mediators in IH neurons through targeting the HIF1α/mtROS pathway.

### Microglial exosomal miR-146a-5p alleviated neuronal inflammation in mice exposed to IH

**Fig. 7:**
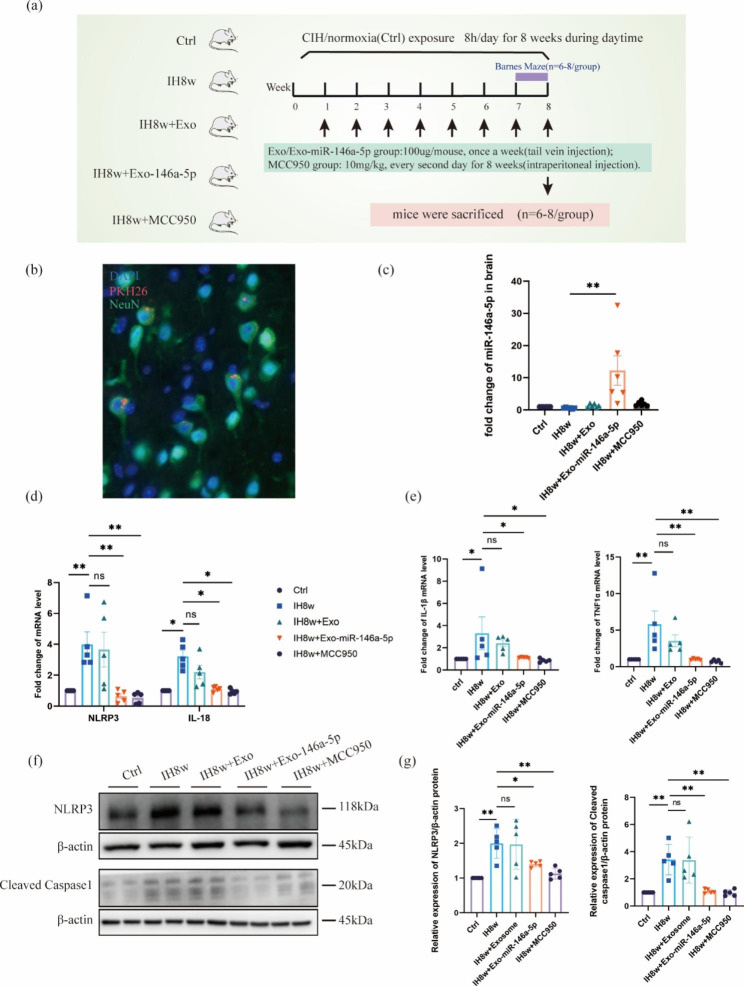
Increased miR-146a-5p in microglial exosomes inhibit neuroinflammation in mice exposed to IH8w. (**a**) Schematic diagram of experimental design. (**b**) Immunofluorescence staining showed that the co-expression PKH26 and NeuN, indicating that the exogenous exosomes were absorbed by neurons of mice treated with IH8w. (**c**) The expression levels of miR-146a-5p in the brain of control, IH8w, IH8w + Exosome, IH8w + Exo-miR-146a and IH8w + MCC950 mice were detected by RT-PCR. (**d-e**) The mRNA expression levels of NLRP3, IL-18, IL-1β, TNFα in the brain of different groups of mice were determined by qRT-PCR. (**f-g**) The protein expression of NLRP3, cleaved caspsase1 in the brain of five groups of mice were performed by western blotting. Data are presented as mean ± SD (n = 4–8) and analyzed using one-way ANOVA with Tukey’s post hoc test or Dunnett’s T3 post hoc test. *P < 0.05, **P < 0.01, and ***P < 0.001

In order to further explore the effects of overexpression of miR-146a-5p in microglial exosomes on NLRP3 inflammasome and neuroinflammation in mice with IH, we divided the experiment into five groups (Fig. 7a). Immunofluorescence staining showed that PKH26 was co-localized with NeuN, which proved that exosomes could be taken up by neurons after injection of exosomes into the tail vein of mice (Fig. 7b). Furthermore, the miR-146a-5p levels were significantly increased in the brain tissue of mice with IH8w after the administration of miR-146a-5p-upregulated microglial exosomes, indicating the success of the model (Fig. 7c). Further studies indicated that NLRP3, IL-18 mRNA levels of the mice treating with IH8w were increased markedly compared with the control group, while their levels were inhibited significantly within the Exo-miR-146a-5p or MCC950 treatment group, and there was no difference in the IH8w + Exo group compared with the IH8w group. (Fig. 7d). Similarly, compared with the control group, the mRNA levels of inflammatory cytokines (IL-1β, TNFα) in the IH8w mice were increased, and their levels were inhibited significantly within the Exo-miR-146a-5p or MCC950 treatment group (Fig. 7e). And the increase of NLRP3, Cleaved Caspase1 protein after IH8w were also significantly reduced by treatment with Exo-miR-146a-5p or MCC950 (Fig. 7f-g). These results indicated that neuroinflammation of mice caused by IH could be alleviated by miR-146a-5p and MCC950.

### Microglial exosomal miR-146a-5p alleviated persistent cognitive deficits in mice induced by IH

**Fig. 8:**
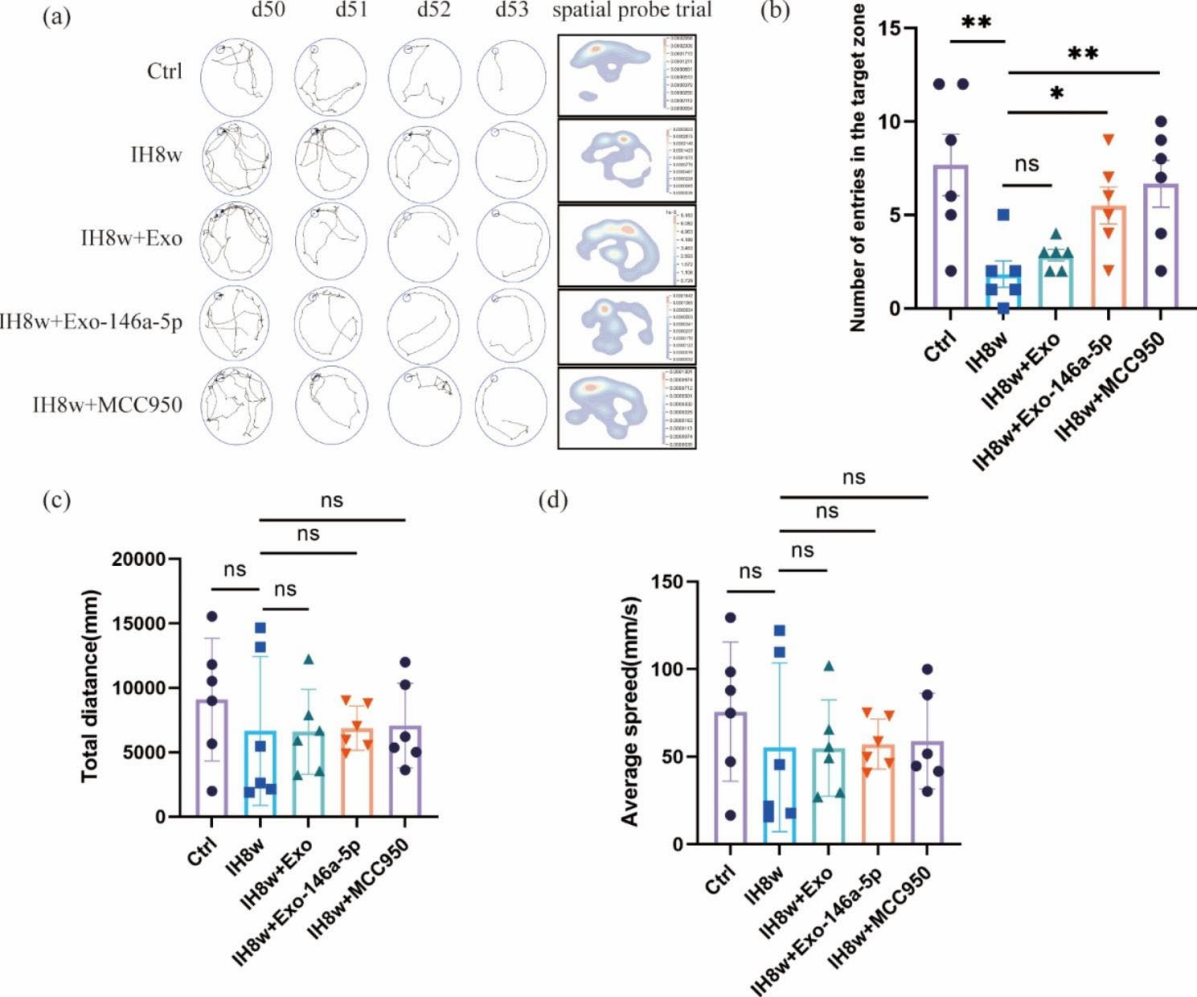
Overexpression of miR-146a-5p in microglial exosomes attenuates memory deficits induced by IH8w in mice. (**a**) The Barnes Maze experiment was conducted on days 50–53, representing training and spatial probe paths of mice in the five groups. (**b**) The number of escape tunnel crossings during the probe trial of Barnes Maze. (**c-d**) The average speed and total distance in the probe phase of Barnes Maze. Data are presented as mean ± SD (n = 4–8) and analyzed using one-way ANOVA with Dunnett’s post hoc test or Dunnett’s T3 post hoc test. *P < 0.05, **P < 0.01, and ***P < 0.001

CIH significantly promoted the spread of pathological tau in P301S mice [4], and Dewachter et al. confirmed that the activation of NLRP3-ASC inflammasome contributed to the spread of tau pathology [34], which is an important cause of cognitive deficits. Therefore, we investigated whether miR-146a-5p could affect the cognitive impairment after IH, and we found that spatial learning and memory capacity could be improved in mice exposed to IH8w after treating with Exo-miR-146a-5p or MCC950, as evidenced by the number of escape tunnel crossings (Fig. 8a-b). The average speed and total distance were found no significant difference between the five groups of mice (Fig. 8c-d). Taken together, the cognitive deficits caused by IH could be attenuated by treating with Exo-miR-146a-5p or MCC950. These results suggest that inhibition of NLRP3 inflammasome may be an essential mechanism in alleviating cognitive impairment caused by IH.

## Discussion

With the increasing prevalence of OSA and the occurrence of long-term complications, especially the neurological damage caused by OSA, we are increasingly concerned about this disease. Positive airway pressure (PAP) therapy remains a critical option for patients with OSA, and long-term use of continuous PAP (CPAP) therapy may improve OSA related cognitive impairment [35], but this research needs to be supported by larger cohort studies. Studies have shown that activation of NLRP3 inflammasome contributed to tau pathology and promoted Alzheimer’s disease progression, and inhibition of NLRP3 inflammasome with MCC950 improved cognitive decline after stroke in diabetic animals [21, 22, 36].

**Fig. 9:**
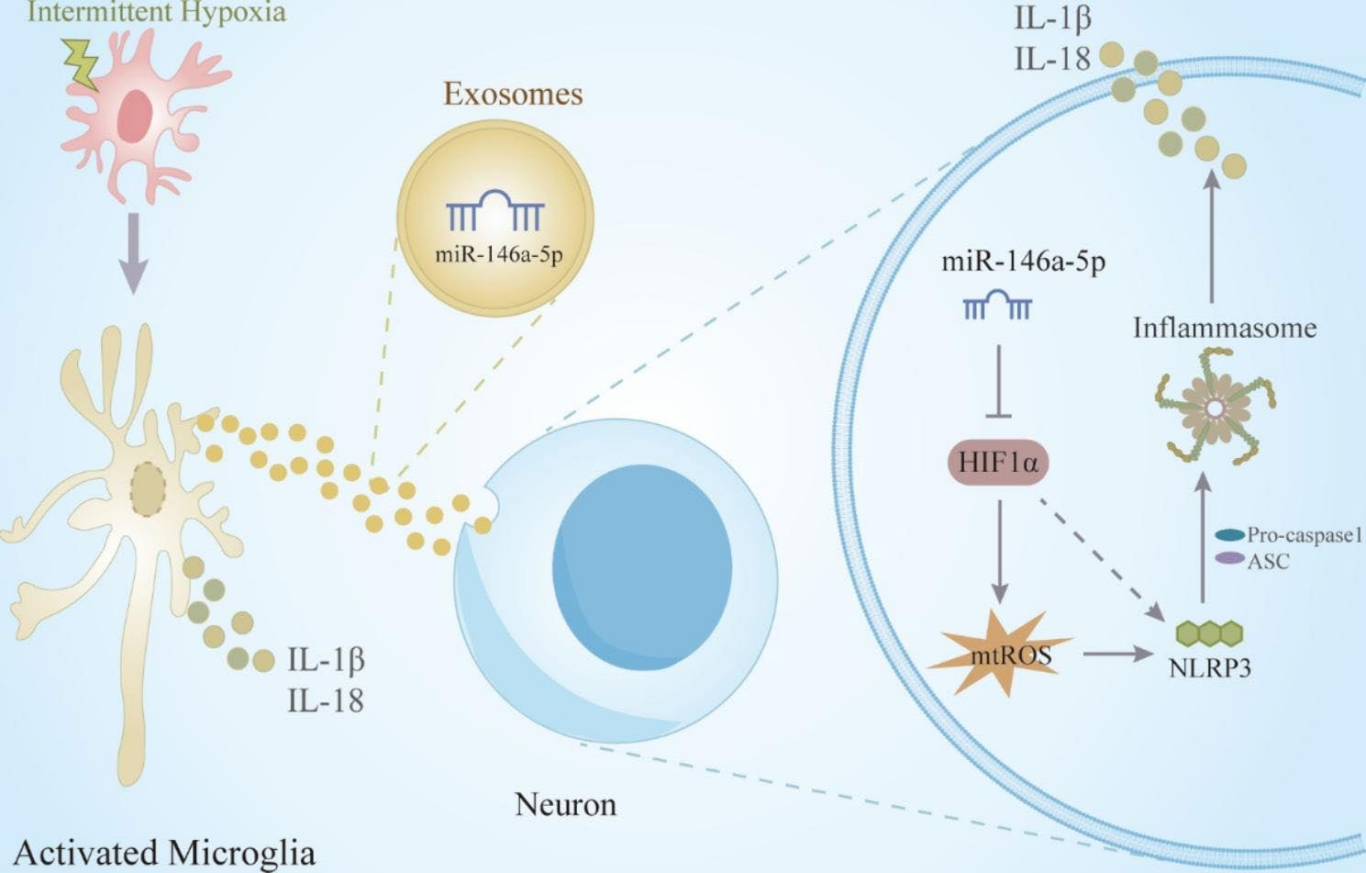
The schematic illustration of upregulated miR-146a-5p in microglial exosomes improving neuroinflammation after IH by targeting the regulation of HIFa/mtROS pathway

Considering the crucial role of NLRP3 inflammasomes in cognitive impairment, our study revealed a correlation between the NLRP3 inflammasome and cognitive impairment following IH. Furthermore, we aimed to elucidate the impact and mechanism of microglial exosomes in IH-induced cognitive deficits by modulating neuroinflammation. We found that: (1) The NLRP3 inflammasome and the secretion of inflammatory factors were significantly activated after IH for 8 weeks, which may be related to microglia. And the number and morphology of microglia were changed and p-tau pathology was also observed in mice with IH8w. (2) The level of miR-146a-5p exhibited a fluctuating trend in microglial exosomes of mice following treatment with IH. (3) Microglial exosome miR-146a-5p regulates neuronal mtROS by targeting HIF1α, and directly or indirectly affects neuroinflammation. (4) Inhibition of NLRP3 inflammasome contributes to the alleviation of neuroinflammation in mice, leading to improve cognitive outcomes (Fig. 9).

In 2013, the crucial involvement of NLRP3 inflammasome in the pathogenesis of AD was revealed, highlighting its role in regulating Aβ deposition and spatial memory in mice [36]. Further, researches on the role of NLRP3 inflammasome in cognitive deficits is growing, although more studies have been conducted on microglia [37]. However, NLRP3 inflammasome was expressed in a variety of cells, including astrocytes [38], endothelial cells [39], neutrophils and monocytes [20]. Moreover, our previous study has confirmed the promotion of neuronal pyroptosis under conditions of sleep deprivation [40]. Similarly, the assembly and activation of the NLRP3 inflammasome in dopamine neurons is also an important strategy to prevent the degeneration of neurons. The deletion of parkin induces mitochondria-derived reactive oxygen species (mtROS) through the accumulation of another parkin ubiquitination substrate, ZNF746/PARIS, which promotes the assembly of neuronal NLRP3 inflammasome complex [6]. Therefore, the NLRP3 inflammasome complex of neurons deserves further study. We demonstrated the increased expression of NLRP3 inflammasome in neurons of mice exposed to IH for 8 weeks, thereby enriching the research on the correlation between neuroinflammation and IH.

In the last decade, numerous studies have focused on the role of extracellular vesicles as carriers of bioactive molecules in the exchange of information between nerve cells, thereby affecting neuroplasticity and cognitive function in physiological and pathological states [41, 42]. Accumulating studies have provided evidence that exosomes can facilitate the tau propagation [12, 43], promoting the progression of the disease. Additionally, exosomes have been shown to promote the clearance of pathological proteins by microglia [44], and the beneficial components contained in exosomes may have neuroprotective effects [18]. Similarly, the involvement of exosomes and exosome-containing cargo in the development and progression of atherosclerosis associated with OSA has been studied [45, 46]. Importantly, exosomes derived from mesenchymal stem cells have protective effects in various organ systems and have specific advantages, including minimal immunogenicity, low risks of tumorigenicity, and limited heterogeneity [47]. Their biological components are transmitted to recipient cells to improve biological function [48]. Therefore, loading different biological components into exosomes and mass production will be a key step in clinical transformation.

miRNAs, the small endogenous RNAs, regulate gene expression post transcriptionally. miRNA is an important regulator of gene expression and a promising candidate molecule for biomarkers, so the study of miRNA still has a great prospect. Researchers specifically analyzed miRNA in microglial EVs of leech [49]and found that miR-146a-5p was expressed in microglia EVs. Similarly, some studies indicated that miR-146a-5p was specific to microglia or mainly derived from microglia, and the deletion of miR-146a could affect function and proteome of microglia [50, 51]. We know that miR-146a-5p is an inflammation-related miRNA, which plays an indispensable role in regulating the pathology of AD and the development of depression [27, 52, 53]. However, other studies have indicated that miRNA overexpression does not seem to induce cognitive deficits in wild mice [54]. In contrast, a multiple miRNAs signature, including miR-146a-5p, was associated with cognitive decline in mouse models for dementia and could be used as a target for RNA therapeutics [28]. Furthermore, Cui et al. found that intranasal administration of miR-146a-5p could reduce the hippocampal pathology and rescue cognitive impairment in the AD mouse model [55]. Therefore, the role of miRNA in cognitive impairment is mixed, which may be related to different disease states. Studies have also shown that miR-146a-5p can reduce intestinal injury in the Necrotizing enterocolitis (NEC) and cognitive decline after surgical trauma by inhibiting inflammation in mice [29, 56]. To sum up, there are still many controversies about the research of miRNA. We have demonstrated that miR-146a-5p in microglial exosomes offers the potential to reduce neuroinflammation and exert neuroprotective effects by targeting HIF1a /mtROS pathway, which provides valuable insights for future clinical studies on IH.

HIF-1 and HIF-2 belong to the HIF family of transcriptional activators, and their activation mediates physiological adaptation to hypoxia. Reportedly, HIF1α could regulate ROS production, neuronal apoptosis and spatial memory ability damage under hypoxia [31, 57]. HIF1 mediated ROS production is partly caused by NADPH oxidases (NOXs) NOX4 mRNA activation [58]. And the elevated ROS downregulates the GluN1, an obligatory subunit of the N-methyl d-aspartate receptor (NMDAR), impairing synaptic physiology and spatial memory function [58]. In the present study, we found an increase of mtROS in neuron after treated with IH, as well as the NLRP3 inflammatory, suggesting that mtROS levels may be associated with NLRP3 inflammatory production, which in turn promote inflammatory responses and impair cognitive function. The following research supports these findings. The pathogenesis of cognitive decline caused by intermittent hypoxia includes oxidative stress, neuroinflammation, cerebral vasculature remodeling, and neural cell death, as well as may also be secondary to daytime sleepiness [31, 58, 59]. Previous studies have also shown that mtROS are closely related to the activation of NLRP3 inflammasome in postoperative cognitive dysfunction and pulmonary fibrosis [60, 61]. And antioxidant was an effective treatment for IH-induced neuropathology [62]. In short, while advancing previous studies, our study is the first to apply an inhibitor of NLRP3 inflammasome to a mice model of intermittent hypoxic, providing effective data for improving memory function. Moreover, NLRP3 deficiency in microglia could reduce neuroinflammation caused by intermittent hypoxia through enhancing mitophagy [30]. In a mouse model of stroke, MCC950 may ameliorate blood-brain barrier dysfunction and brain edema by acting on microglia and Gr-1 + myeloid cells [20]. Therefore, further investigation is required to elucidate the mechanism by which the NLRP3 inflammasome impacts p-tau protein and cognitive function.

As a kind of classic inflammatory cells in the brain, activated microglial cells will release inflammatory cytokines, which cause the onset of neuroinflammation. NLRP3 inflammasome is also expressed in neurons, and the inflammation around them may be secreted by microglia cells or self-generated by neurons. The levels of inflammatory cytokines secreted by neurons hold great prospects for research [6]. So far, it seems impossible to distinguish whether inflammatory cytokines in brain tissue are produced by microglia, neurons or other nerve cells. Levels of neuronal inflammatory cytokines have been studied in traumatic brain injury and sleep deprivation [40, 63]. We cannot deny that miR-146a-5p affected the secretion of inflammatory cytokines in microglia cells. As indicated in our study, miR-146a-5p alleviates the reactive microglia. The decrease of neuroinflammation in mice with intermittent hypoxia after the administration of microglial exosomes with miR-146a-5p overexpression may be partly directly caused by the decrease of inflammatory cytokines secreted by microglia, and the effect of miR-146a-5p-containing exosomes secreted by microglia on neurons to regulate neuronal inflammation is also an important link.

Inevitably, there may still be many problems with our IH model, such as: (1) Because the OSA models must be induced [64], and obesity cannot be taken into account. However, Clinically, OSA patients are clearly associated with obesity, which may be the difference between natural and induced OSA; (2) Since the mice are all nocturnal, IH mice were induced during the daytime to simulate human OSA. However, it should be noted that IH during the daytime can induce sleep deprivation to some extent [65], which might potentially affect tau pathology to a certain degree [65].

Previous studies have demonstrated that miR-146a-5p inhibited intestinal inflammation [29], which is consistent with our results. NLRP3 inflammasome is a key molecule affecting pyroptosis. Also, autophagy and apoptosis play important roles in regulating cognitive impairment caused by IH [66, 67]. However, other programmed cell death pathways in OSA patients, including ferroptosis, necroptosis, remain to be further investigated [68, 69]. Furthermore, the predominant mode of cell death has not yet been studied, and it is possible that a combination of multiple death modes may be the focus of our future research.

## Conclusions

According to this study, it is demonstrated that NLRP3 inflammasome and inflammatory cytokines are elevated in mice treated with IH for 8 weeks, as well as microglial activation. Increased miR-146a-5p levels in microglia improved NLRP3 inflammasome and inflammatory factors by targeting the regulation of HIF1α/mtROS pathway, and inhibition of NLRP3 inflammasome alleviated cognitive impairment of mice exposed to IH. From these studies, we can gain a deeper understanding the relationship between NLRP3 inflammasomes and the neurological deficiency induced by IH. This knowledge helps us identify appropriate therapeutic targets and interventions, opening up avenues for clinical intervention.

## Methods and materials

### Animals

Adult Male C57BL/6J mice aged 8–10 weeks old were obtained from the Chinese Academy of Military Sciences (Beijing, China) and had free access to food and water with a comfortable environment. All protocols were approved by the Animal Care and Use Committee of Tianjin Medical University and were performed according to the National Institutes of Health Guide for the Care and Use of Laboratory Animals.

### IH exposure (mice)

IH exposure paradigm is shown in Fig. 1a. Briefly, the mice were placed in a chamber with inhaled oxygen concentration alternating between 21% and 5% at comfortable temperature(22–24 ℃) and humidity (less than 50%) [70]. Mice were exposed to IH for 8 h per day, 40 cycles per hour. The control mice were placed in the normoxia chamber (21% O_2_) for 8 h per day at the same time [4, 71]. During the rest of the time, the mice (normoxia and IH) were placed in room air, and free received a 12:12 h light-dark cycle.

### Experimental design and treatment

In order to evaluate whether miR-146a-5p in microglial exosome affects the NLRP3 inflammasome, neuroinflammation and behavioral defects in mice. We divided the mice into five groups: Control (Ctrl), IH8w (IH8w), IH8w treated with unedited microglial exosomes (IH8w + Exo), IH8w treated with over-expression of miR-146a-5p in microglial exosomes (IH8w + Exo-miR-146a), IH8w treated with MCC950(IH8w + MCC950).

Mice treated with exosomes were administered 100ul PBS containing 100ug Exos via tail vein once a week for 8 weeks [72, 73]. MCC950(S8930, Selleck), a selective, small-molecule NLRP3 inflammasome inhibitor was dissolved in DMSO and diluted by 5% DMSO, 40% PEG 300, 5% Tween 80, and 50% ddH_2_O. Mice in the MCC950 group was injected intraperitoneally at a dose of 10 mg/kg once every two days for 8 weeks [20, 74], and the Barnes Maze experiment was performed during the eighth week of treatment. The detailed experimental design of animal studies is shown in Fig. 6(a).

### Behavioral test

The Barnes maze test was conducted during the eighth week of IH (Fig. 6(a)), and one adaptation was carried out before the start, followed by four consecutive days of training phase (4 trials per mouse on days 50–53 after IH). After that, the escape tunnel was removed for detection, and mice were free to explore for 2 min. All behaviors were videotaped and analyzed [75, 76]. The escape latency and the number of escape tunnel crossings were calculated to assess spatial learning and memory abilities of the mice.

### BV2 microglia culture

BV2 microglia cell lines were purchased from Nankai University and cultured in DMEM/F12 medium supplemented with 10% Exosome-free fetal bovine serum (FBS), 100 U/mL penicillin, 100 µg/mL streptomycin (Gibco, Gaithersburg, MD, USA) at 37 °C under 5% CO^2^. The purity was determined by immunofluorescence staining using Iba1.

### miR-146a-5p mimics transfection

In order to further study the function of miR-146a-5p in microglial exosomes, miR-146a-5p mimics (GenePharma, Shanghai, China; Table 1) were transfected into microglia. In brief, the miR-146a-5p mimics dry powder was dissolved in DEPC water according to the instructions. 5ul miR-146a-5p mimics and 5ul Lipofectamine® RNAiMAX (invitrogen, Carlsbad, CA, USA) were prepared in 150ul DMEM medium, respectively. Then the diluted miR-146a-5p mimics were added to the diluted Lipofectamine® RNAiMAX. The mixture was mixed well and incubated for 10 min. The above mixture was added to the 6-well plates in DMEM/F12 medium with 10% FBS, and incubated in the incubator for 24-48 h. After that, IH treatment or detection was performed.

**Table 1:** Sequences of miR-146a-5p mimics and siRNA oligomers for transfection

Gene	Sequence, 5’-3’	
	S	AS
siNLRP3	GCUGGAAUCUCUCCACAAUTT	AUUGUGGAGAGAUUCCAGCTT
miR-146a-5p mimics	UGAGAACUGAAUUCCAUGGGUU	CCCAUGGAAUUCAGUUCUCAUU
Negative control	UUCUCCGAACGUGUCACGUTT	ACGUGACACGUUCGGAGAATT

### Isolation of microglial exosomes from brain of mice

The mice were sacrificed at 2, 5, 8 weeks after IH by transcardiac perfusion with PBS. Then, the hippocampus and cortex were detached to extract microglial exosomes [18]. In Brief, the tissue was digested with papain (Solarbio, Beijing, China) and then centrifuged at 4 °C for 10 min at 2000 g to remove cell debris. Further removal of cell particles was performed by centrifugation for 30 min at 10,000 g at 4 °C. Next, the supernatant was filtered through a 0.22 μm filter to remove dead cells and large particles.

After ultracentrifugation for 120 min at 100,000 g at 4 °C, the supernatant was removed, and the pellets were re-suspended in 350ul calcium- and magne-sium-free Dulbecco’s PBS (Thermo Fisher Scientific) and incubated for 60 min at room temperature with 1.5 mg rat anti-mouse CD11b bitinylated antibody (Thermo Fisher Scientific) in 50 µL of 3% BSA, followed by addition of 10 µl of Pierce Streptavidin Plus UltraLink Resin (Thermo Fisher Scientific) in 40 µL of 3% BSA and incubation for 30 min at room temperature with mixing. After centrifugation for 10 min at 800 g at 4℃, the supernatant was removed, and the pellets was suspended in 100µL of cold 0.05 M glycine- HCl (pH 3.0), mixed for 10 s [18]. After centrifugation for 10 min at 4000 g at 4℃, the supernatant was retained and stored for a short time at 4 ℃ for the next experiment.

### Microglial exosome extraction and identification

The microglia medium was collected into 50ml polypropylene tubes, and centrifuged at 4 °C at 300 g for 10 min to remove free cells, and then the supernatant was transferred to a new centrifuge tube, and centrifuged at 4 °C for 10 min at 2000 g to remove cell debris. Removal of cell particles was further performed by centrifugation for 30 min at 10,000 g at 4 °C. Next, the supernatant was filtered through a 0.22 μm filter to remove dead cells and large particles. After ultracentrifugation for 120 min at 100,000 g, the supernatant was removed, and the pellet was re-suspended with an appropriate amount of PBS and then stored at 4 ℃ for a short time for the next experiment.

For exosome identification, the morphology of particles was observed by transmission electron microscopy (TEM, HT7700; Hitachi, Tokyo, Japan). In short, the diluted particles were mixed with the same amount of 4% paraformaldehyde, and then 20ul samples were added onto the glow-discharged, carbon-coated formvar film that attached to a metal specimen grid. The grid was incubated with 1% glutaraldehyde for 5 min at room temperature, and washed with distilled water. Then it was dried with a filter paper for 20–30 min, and an equal amount of 10% uranyl acetate was added to the grid for 5 min at room temperature, and then an appropriate amount of methyl cellulose - uranyl acetate was added and stored at 4 °C for 10 min. The solution is then sucked dry and the sample is viewed with TEM. Size distribution of particles was tested by Nano Particle Tracking and Zeta Potential Distribution Analyzer according to manufacturer’s instructions. Biomarkers of exosomes were identified by immunoblot analysis.

### HT22 hippocampal neuron and primary neuron culture

HT22 hippocampal neuron cells were obtained from infrastructure cell line resources in China and cultured in high glucose DMEM medium with 5% Exosome-free fetal bovine serum, appropriate amounts of penicillin and streptomycin.

Primary neurons were obtained from the brains of newborn mice [63]. The mice were euthanized by cervical dislocation, and the brain tissue was extracted in Ice Dulbecco-Hanks’ solution. After stripping meninges, blood vessels, and cortex, the hippocampal tissue was minced and digested in DMEM medium with 0.25% trypsin and DNase for 15 min at 37℃, then centrifuged and resuscitated with an appropriate amount of complete medium. The cell suspensions were planted in petri dishes coated with poly-D-lysine in advance. After four hours, the special neurobasal medium with 2% B27, 100 U/mL penicillin, 100 µg/mL streptomycin and 1% glutamine were used as the culture medium.

The purity was identified by immunofluorescence of microtubule-associated protein2 (MAP2). After seven days of culture, the neurons were treated with IH or/and exosomes.

### Intermittent hypoxia model (cells)

The above cultured cells were replaced with medium as needed before intermittent hypoxia, and cells were maintained in M6 plates under two different conditions: normoxia and intermittent hypoxia. Cells cultured in normoxia were placed under standard conditions (37 °C, 21% O_2_, 5% CO_2_), and cells cultured in IH were exposed to a hypoxic chamber that maintained cycles of normoxia (37 °C, 21% O_2_, 5% CO_2_, 10 min) and hypoxia (37 °C, 1% O_2_, 5% CO_2_, 5 min) for 12 h [7, 77].

### Treatment of neurons with microglial exosomes

For exosomes-treated neurons, 100ug exosomes were added when the density of cultured neurons was 50–60%, and IH treatment was performed on the second day, followed by qRT-PCR or WB experiments [72]. Neurons were randomly allocated into one of four groups: control treated with unedited microglial exosome (Ctrl + Exo), control treated with over-expression of miR-146a-5p in microglial exosome (Ctrl + Exo-146a), IH treated with unedited microglial exosome (IH + Exo), IH treated with overexpressing miRNA microglial exosome (IH + Exo-146a).

### Exosome fluorescent label

Exosomes are marked with PKH26 (red, Sigma-Aldrich, USA) according to manufacturer’s protocol. In Brief, 4 µl PKH26 dye was added into 1ml diluent C, and the extracted exosomes were mixed with the above solution and incubated at 37℃ for 10 min. The labelling reaction was stopped when 1% BSA was added. The labeled exosomes were super-centrifuged at 100,000 g for 120 min and the pellet were diluted in PBS for further experiments.

### The plasmid and miRNA transfection

Hypoxia-inducing factor-1α (HIF-1α) plasmids and the control empty plasmid vector (GenePharma, Shanghai, China) were transfected into cells using jetPRIME® (Polyplus-transfection S.A, Illkirch, France) according to the manufacturer’s instructions. 2ug DNA plasmid was mixed with 5ul miR-146a-5p mimics in 200 µL of jetPRIME® buffer, and 4 µL jetPRIME® reagent was added to the above solution, vortex for 1 s, spin down briefly, and incubated for 15 min at room temperature. Then, the transfection mix was added to the 6-well plates in the serum-containing medium dropwise, and incubated in an incubator for 24-48 h for further experiments.

### The siNLRP3 transfection

The transfection of siNLRP3 (a specific RNA silencer for NLRP3; Table 1) was performed as described above. The siNLRP3 dry powder was dissolved in DEPC water according to the instructions. 5ul siNLRP3 and 5ul Lipofectamine® RNAiMAX (invitrogen, Carlsbad, CA, USA) were prepared in 150ul DMEM medium, respectively. Then the diluted siNLRP3 were added to the diluted Lipofectamine® RNAiMAX. The mixture was mixed well and incubated for 10 min. The above mixture was added to the 6-well plates in DMEM medium with 10% FBS, and incubated in the incubator for 24-48 h. After that, IH treatment or detection was performed.

### Caspase1 inhibition assays

HT22 hippocampal neurons was treated with 30 µg/mL Ac-YVAD-cmk (GC42721, GLPBIO) for 16 h [7].

### RNA extraction and quantitative real-time polymerase chain reaction (qRT-PCR)

Total RNA was extracted from cultured cells or brain tissue using the TransZol Up Plus RNA Kit (TransGEN, Beijing, China) following the manufacturer’s instructions after 24–48 h of transfection.

RNA concentration and quality was measured by Nanodrop Spectrophotometer (Thermo Scientific, Waltham, MA, USA).

Reverse transcription and RT-PCR (miRNA) were performed using the Hairpin-itTM miRNAs RT-PCR Quantitation Kit (GenePharma, Shanghai, China) and with special primers following the manufacturer’s instructions. Reverse transcription and qRT-PCR (mRNA) were performed with corresponding primers using the *TransScript*® One-Step gDNA Removal and cDNA Synthesis SuperMix (AT311, TransGEN, Beijing, China) and *PerfectStart*® Green qPCR SuperMix (AQ601, TransGEN, Beijing, China) respectively. The relative value of miRNA/mRNA transcription was calculated using the 2^−∆∆CT formula, and U6/GAPDH was used as the internal control for normalization (Table 2).

**Table 2:** Primer sequences of miRNA and mRNAs oligomers for RT-PCR.

Gene	Primer sequence, 5’-3’	
	Forward	Reverse
miR-146a-5p	TAATCGTGTGAGAACTGAATTCCA	TATGGTTTTGACGACTGTGTGAT
U6	CAGCACATATACTAAAATTGGAACG	ACGAATTTGCGTGTCATCC
NLRP3	GCCGTCTACGTCTTCTTCCTTTCC	CATCCGCAGCCAGTGAACAGAG
IL-18	AGACCTGGAATCAGACAACTTT	TCAGTCATATCCTCGAACACAG
HIF1α	TTTCTCAGTCGACACAGCCT	AATTGAGCGGCCCAAAAGTT
IL-1β	ATGGGCTGGACTGTTTCTAATGC	TCTTGTGACCCTGAGCGACC
TNFα	CGGCATGGATCTCAAAGACAAC	GAAGAGAACCTGGGAGTAGACAAG
GAPDH	GCCAAGGCTGTGGGCAAGGT	TCTCCAGGCGGCACGCAGA

### Detection of mitochondrial reactive oxygen species

5 μm of MitoSOX^™^ Red (M36008, Invitrogen, Carlsbad, CA, USA) staining solution was added to the HT22 cells in the culture plate and incubated for 10 min at 37 °C away from light. Then, the cells were washed with warm PBS three times. Nuclei were stained with Hoechst 33,342 (C1027, Beyotime, China). Images were photographed using an OLYMPUS confocal microscope (Tokyo, Japan).

### Immunofluorescence (IF)

The cells were fixed with 4% paraformaldehyde (PFA), and the mice were killed by transcardial perfusion of cold phosphate-buffered saline (PBS) and 4% PFA. Subsequently, the brain tissue was removed completely and fixed with 4% PFA overnight. After gradient dehydration with sucrose, the brain was embedded within the optimal cutting temperature (Sakura, Torrance, CA, USA). The brain samples were cut into slices of appropriate thickness using a -20 °C frozen slicer for IF staining.

The cells or brain tissue sections were placed at room temperature for 15 min from the − 20℃ refrigerator, washed with PBS for three times, treated with 0.3%Triton for 30 min at room temperature, and then incubated with 3% BSA for 60 min. They were then incubated with primary antibodies (Table 3) overnight at 4℃. On the second day, the sections were incubated with secondary antibody for one hour at room temperature after washing with PBS, and then DAPI (Abcam, UK) was used to stain the nucleus.

**Table 3:** List of the primary antibodies used in this study

Antibody	Catalogue number	Brand	Dilution	MW (kDa)	Application
Iba1	A20844	Abclonal	1:100	NA	IF
MAP2	sc-74,421	Santa Cruz	1:100	NA	IF
NeuN	Ab177487	abcam	1:500	NA	IF
iNOS	13,120	CST	1:200	NA	IF
NLRP3	MAB7578	R&D system	12.5ug/ml	NA	IF
NLRP3	ab263899	abcam	1:1000	118	WB
Cleaved Caspase1	67,314	CST	1:1000	20/22	WB
GSDMD	Ab209845	abcam	1:1000	32/53	WB
ASC	67,824	CST	1:1000	22	WB
CD63	ab134045	abcam	1:1000	30–65	WB
Alix	2171	CST	1:1000	95	WB
TSG101	72,312	CST	1:1000	50	WB
HIF1α	14,179	CST	1:1000	120	WB
p-tau	9632	CST	1:1000	50–80	WB
p-tau	49,561	CST	1:200	NA	IF
β-actin	4970	CST	1:1000	45	WB

Abcam: Abcam, Cambridge, MA, USA; CST: Cell Signaling Technology, Danvers, MA, USA; MW: molecular weight; NA: not applicable; Santa Cruz: Santa Cruz Biotechnology, Santa Cruz, CA, USA; R&D system: R&D system,USA; Abclonal:Abclonal, China; WB: western blot; IF: immunofluorescence.

### Immunoblotting for protein evaluation

Western blotting for NLRP3, Cleaved Caspase1, GSDMD, ASC, CD63, Alix, TSG101, HIF1α, p-tau, β-actin was performed, as a previous description [18, 78] (Table 3). The band gray values were measured with ImageJ (National Institutes of health, Bethesda, MD, USA).

### Statistical analysis

SPSS 27.0 and GraphPad Prism 9.4 were used for statistical analysis and mapping of the data. All data were represented as mean ± standard deviation (SD). One-way ANOVA followed by LSD post hoc test, Dunnett’s post hoc test, Tukey’s post hoc test or Dunnett’s T3 post hoc test were used for comparisons of multiple groups, and student’s t test were used for comparisons of two groups. The P value < 0.05 was considered statistically significant.

## Electronic supplementary material

Below is the link to the electronic supplementary material.

Supplementary Material 1: Figure S1: Unedited microglial exosomes had no effect on the NLRP3 inflammasome in IH neuron.

Supplementary Material 2: Figure S2: Detection of transfection efficiency in neurons transfected with overexpressed HIF1α plasmids.

## Data Availability

All data generated or analysed during this study are included in this published article; the further datasets are available from the corresponding authors upon reasonable request.

## List of abbreviations

AD: Alzheimer’s disease
ANOVA: Analysis of variance
CIH: Chronic intermittent hypoxia
DG: Dentate gyrus
EVs: Extracellular vesicles
FBS: Fetal bovine serum
IH: Intermittent hypoxia
MAP2: Microtubule-associated protein2
mtROS: Mitochondrial Reactive oxygen species
NLRP3: Nucleotide-binding oligomerization domain-like receptor 3
NTA: Nanoparticle tracking analysis
OSA: Obstructive sleep apnea
p-tau: Phospho-tau
qRT-PCR: Quantitative Real-Time Polymerase Chain Reaction
SD: Standard deviation
TEM: Transmission electron microscopy
