# Supplementary material for: Microglial exosomes alleviate intermittent hypoxia-induced cognitive deficits by suppressing NLRP3 inflammasome
Source: Biol Direct. 2023 Jun 13;18:29. doi: 10.1186/s13062-023-00387-5 (PMC10262550; doi:10.1186/s13062-023-00387-5)

Fig. S1: Unedited microglial exosomes had no effect on the NLRP3 inflammasome in IH neurons. (a) NLRP3 and cleaved caspase1 protein were measured by immunoblot after 12 hours of IH or/and treatment with unedited exosomes in neurons. (b) Quantitative data of NLRP3 and cleaved caspase1 protein levels after 12 h of IH or/and treatment with unedited exosomes in neurons.


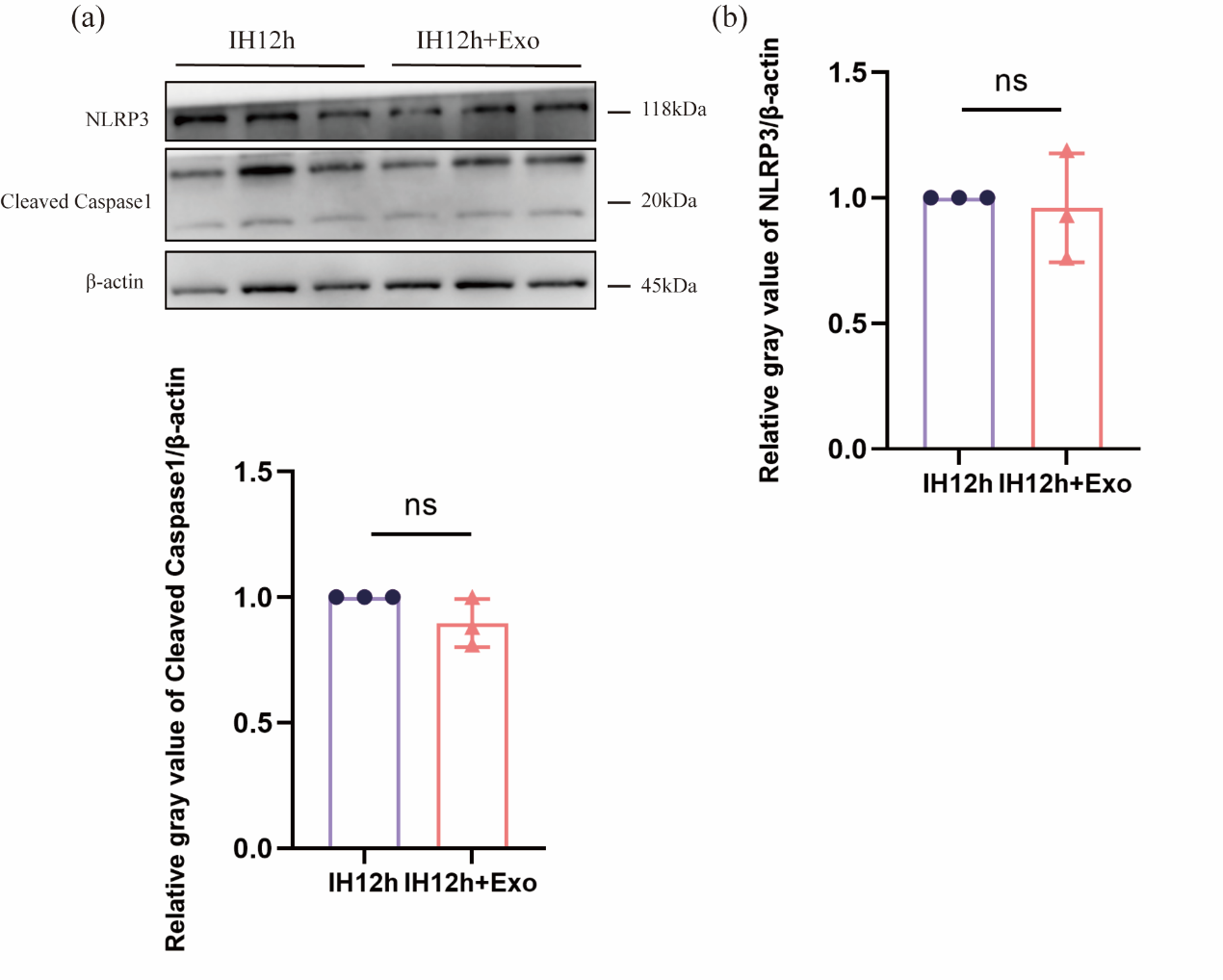

Supplement: Supplementary file 1 — Supplementary Material 1: Figure S1: Unedited microglial exosomes had no effect on the NLRP3 inflammasome in IH neuron. [file 13062_2023_387_MOESM1_ESM.docx]
