# Supplementary material for: Microglial exosomes alleviate intermittent hypoxia-induced cognitive deficits by suppressing NLRP3 inflammasome
Source: Biol Direct. 2023 Jun 13;18:29. doi: 10.1186/s13062-023-00387-5 (PMC10262550; doi:10.1186/s13062-023-00387-5)

Fig. S2: Detection of transfection efficiency in neurons transfected with overexpressed HIF1α plasmids. (a) The level of HIF1α protein in cultured neurons following transfection with overexpressed HIF1α plasmids.


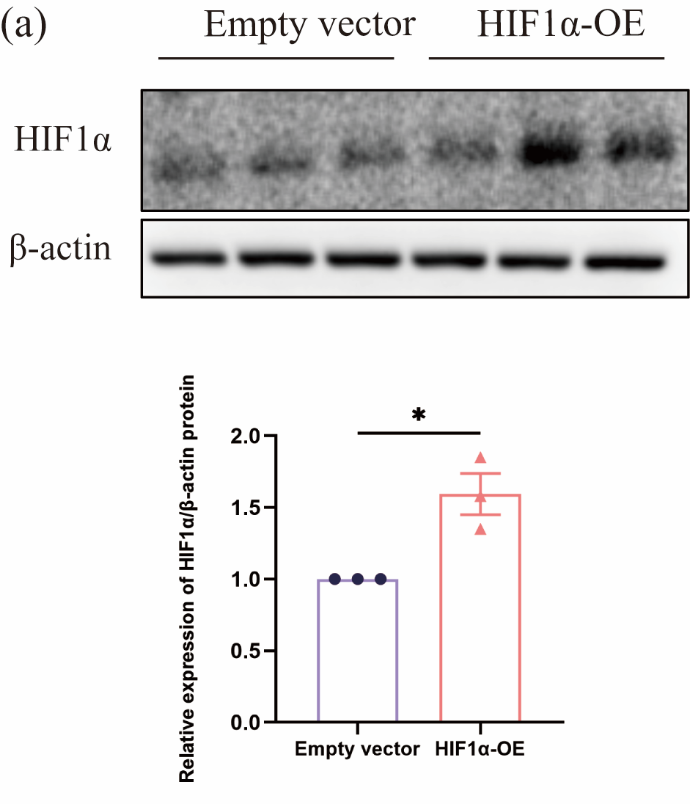

Supplement: Supplementary file 2 — Supplementary Material 2: Figure S2: Detection of transfection efficiency in neurons transfected with overexpressed HIF1α plasmids. [file 13062_2023_387_MOESM2_ESM.docx]
